# Audiovisual integration of speech in a patient with Broca's Aphasia

**DOI:** 10.3389/fpsyg.2015.00435

**Published:** 2015-04-28

**Authors:** Tobias S. Andersen, Randi Starrfelt

**Affiliations:** ^1^Section for Cognitive Systems, Department of Applied Mathematics and Computer Science, Technical University of DenmarkLyngby, Denmark; ^2^Department of Psychology, Center for Visual Cognition, University of CopenhagenCopenhagen, Denmark

**Keywords:** audiovisual, speech perception, aphasia, Broca's area, multisensory integration

## Abstract

Lesions to Broca's area cause aphasia characterized by a severe impairment of the ability to speak, with comparatively intact speech perception. However, some studies have found effects on speech perception under adverse listening conditions, indicating that Broca's area is also involved in speech perception. While these studies have focused on auditory speech perception other studies have shown that Broca's area is activated by visual speech perception. Furthermore, one preliminary report found that a patient with Broca's aphasia did not experience the McGurk illusion suggesting that an intact Broca's area is necessary for audiovisual integration of speech. Here we describe a patient with Broca's aphasia who experienced the McGurk illusion. This indicates that an intact Broca's area is not necessary for audiovisual integration of speech. The McGurk illusions this patient experienced were atypical, which could be due to Broca's area having a more subtle role in audiovisual integration of speech. The McGurk illusions of a control subject with Wernicke's aphasia were, however, also atypical. This indicates that the atypical McGurk illusions were due to deficits in speech processing that are not specific to Broca's aphasia.

## Introduction

Broca's area has long been known to be necessary for speech production as evidenced by the severe impairment to speech production, known as expressive, non-fluent or Broca's aphasia, caused by lesions to this area located in the ventrolateral prefrontal cortex (Broca, 1861). It has long been known that expressive aphasia can be dissociated from impairment of speech perception known as receptive, fluent or Wernicke's aphasia (Wernicke, 1874), caused by damage to Wernicke's area in the posterior superior temporal sulcus (STS). This dissociation may, however, not be entirely complete as speech perception under adverse conditions can be impeded in Broca's aphasics (Blumstein et al., 1977; Moineau et al., 2005). Both Broca's and Wernickes' area are located in the left hemisphere in about 90% of right-handers and 70% of left-handers (Knecht et al., 2000).

A role for Broca's area is also suggested by studies showing that this area is active not only during speech production, but also during speech perception, particularly when it involves lip-reading (Watkins et al., 2003; Ojanen et al., 2005; Skipper et al., 2005). Furthermore, speech perception also activates motor cortex, and this activation is articulator-specific so that labial articulations activate the lip region and lingual articulations activate the tongue region of the motor cortex (Pulvermüller et al., 2006). This effect is causal as shown by studies in which de-activation of premotor (Meister et al., 2007) and primary motor (Möttönen and Watkins, 2009) cortex using TMS impeded speech perception. As changes in the excitability of motor cortex due to speech perception are correlated with activity in Broca's area (Watkins and Paus, 2004) this activation is likely to go via Broca's area (Möttönen and Watkins, 2012).

These findings suggest a role for Broca's area in speech perception partly echoing the claims made by the Motor Theory (MT) of speech perception (Liberman and Mattingly, 1985; Galantucci et al., 2006). According to the MT, in its strongest form (Liberman and Mattingly, 1985), the internal representation of phonemes is not based on auditory templates but instead on the motor commands issued to articulate the phonemes. Hence, according the MT, speech, perceived by hearing or lip-reading (Liberman, 1982), must be mapped onto motor commands, located in the motor system, to be understood.

The MT was fueled by the discovery of mirror neurons in pre-motor area F5 in non-human primates (Gallese et al., 1996). These neurons respond both when performing a goal-directed action and when seeing that action performed by others. As area F5 has been suggested to be the non-human primate homolog of Broca's area in humans (Rizzolatti and Arbib, 1998; Binkofski and Buccino, 2004) this discovery gave rise to the mirror neuron motor theory of speech perception: Mirror neurons in Broca's area may decode auditory and visual speech to articulatory actions and activate their representation in motor areas (Rizzolatti and Craighero, 2004). Several observations support this hypothesis. First, some mirror neurons can respond to hearing as well as seeing an action (Kohler et al., 2002), which seems like a necessary ability for neurons involved in speech perception. Second, mirror-neurons can respond not only to hand movements but also to communicative mouth movements (Ferrari et al., 2003).

Broca's area thus has a central role in audio-visual-motor integration according to the mirror neuron MT of speech perception. The integration of auditory and visual information in speech perception is evidenced by the congruency effect, which is a general facilitation when watching the interlocutor's face (Sumby and Pollack, 1954), and by the McGurk illusion (McGurk and MacDonald, 1976) in which the perception of a clearly perceived phoneme (e.g., /ba/) is altered (e.g., to /da/) when it is perceived dubbed onto an a face articulating an incongruent phoneme (e.g., /ga/). For this integration to occur the auditory and visual information must be mapped onto a common representation, which, according to the MT, is articulatory (Liberman, 1982). On the basis of the mirror neuron MT, Ramachandran and co-workers suggested, in a preliminary report, that Broca's area is necessary for audiovisual integration of speech (Ramachandran et al., 1999). In support of this hypothesis they reported results from a single Broca's aphasic that did not experience the McGurk illusion. This result has, to our knowledge, never been tested more fully, which is what we aim to do in the current study.

The strongest claim of the original MT is that the motor system is essential for speech perception, but even though fairly recent studies have argued for this (Fadiga et al., 2002; Meister et al., 2007), substantial evidence against it has also been reported (Lotto et al., 2009). The resolution may lie in a more complex and nuanced account of the MT in which the motor system is not necessary for speech perception but rather has a supplementary role (Scott et al., 2009).

This complexity is captured in dual-stream anatomical models in which the dorsal and ventral pathways link areas involved in speech perception with areas involved in speech production (Skipper et al., 2005, 2007; Hickok and Poeppel, 2007; Rauschecker and Scott, 2009; Jääskeläinen, 2010). The dorsal stream projects from the posterior part of the STS, containing Wernicke's area, to premotor cortex, which projects further onto Broca's area. According to the classical Wernicke-Geschwind model this stream has a crucial role for feeding perceptual information to speech production centers although more recent evidence suggests it has a more complex role (Bernal and Ardila, 2009). Dual stream models of speech perception have suggested that the dorsal pathway also underlies perceptual processing of audiovisual speech based on articulatory representations (Skipper et al., 2005, 2007; Jääskeläinen, 2010). This suggests an important role for articulatory representations in audiovisual integration of speech, as the posterior STS is the area in the perceptual system that is most consistently found to be activated super-additively by audiovisual speech. The ventral stream projects from the anterior part of the STS to the ventrolateral prefrontal cortex, containing Broca's area (Bernstein and Liebenthal, 2014). Thus, although one dual-stream model hypothesizes that only processing in the dorsal pathway is based on articulatory representations (Skipper et al., 2005, 2007; Jääskeläinen, 2010) the ventral pathway may also be important for sensory-motor integration of speech. The complexity of sensory-motor interactions in this dual-stream network means that dysfunction of parts of the network may lead to subtle and complex effects in agreement with moderate versions of the MT. Yet, as both streams may ultimately project to Broca's area, the dual-stream model does not preclude that this area can have a necessary role in audiovisual integration of speech as suggested by Ramachandran et al. and the strong version of the mirror neuron MT.

The purpose of the current study is to test a patient suffering from Broca's aphasia in a standard auditory, visual, and audiovisual speech perception task. The hypothesis is that the patient should show no sign of audiovisual integration, as measured by the McGurk illusion and the congruency effect, if Broca's area is necessary for audiovisual integration of speech. Alternatively, audiovisual integration could be weak rather than completely eliminated, which would still support a supplementary role for Broca's area in audiovisual integration. As the McGurk illusion is subject to great individual variability, this alternative hypothesis is more difficult to test. Furthermore, as the strength of the McGurk illusion depends not only on the observer but also on the experimental setup (Magnotti and Beauchamp, 2014), especially the stimulus material, we also tested a patient with receptive aphasia and two healthy control participants to confirm that our experimental setup could induce the McGurk illusion.

## Methods

### Case reports

#### Patient ML: Broca's aphasia

**Figure 1:**
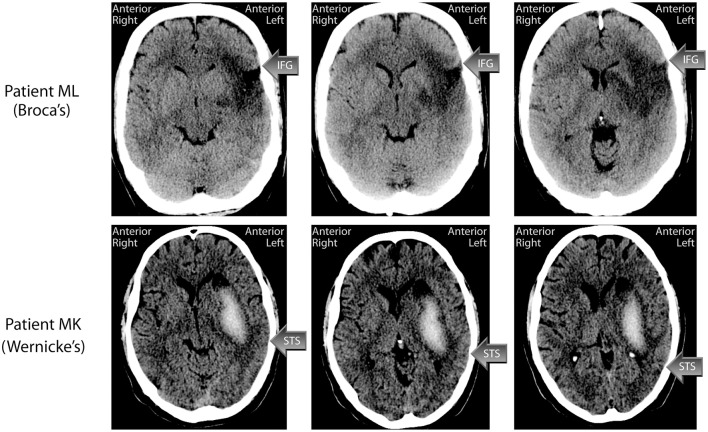
**CT-scans of the lesions of patients ML (Broca's aphasic) and MK (Wernicke's aphasic)**. Arrows indicate the approximate location of the inferior frontal gyrus (IFG) in Patient ML and the superior temporal sulcus (STS) in Patient MK. See main text for a description of the patients' lesions.

ML is a right-handed, male native Danish speaker, who, at the time of testing was 47 years old. ML suffered an ischemic stroke in 2006, at the age of 42, for which he received trombolysis treatment. The stroke affected the anterior 2/3 of the territory of the Middle Cerebral Artery (MCA) in the left hemisphere, including both cortex and white matter. A computerized tomography (CT) scan (see Figure 1) a week post-stroke, showed that both the inferior and middle frontal gyri were affected, and that there was a smaller cortical affection laterally in the superior frontal gyrus. There was also loss of substance frontotemporally around the Syvian Fissure. The entire insular cortex was affected, and the infarction stretched rostrally in centrum semiovale and medially in the left basal gangila. There was Wallerian degeneration with atrophy of the left cerebral penduncle and left mesencephalon, and a compensatory widening of the left lateral ventricle. The entire inferior frontal gyrus, which contains Broca's area, was thus affected by the lesion.

Initially following his stroke, ML had global aphasia, right hemianopia, and right-sided hemiparesis affecting the arm and leg. Neuropsychological examination 4 months post injury showed that ML was able to say “yes” and “no,” as well as his own name and the name of his two children, but nothing else. At this time, he had severe apraxia of speech, and his receptive language abilities were also affected, while his understanding of gestures was comparatively good. His reading and writing was also severely affected. In 2007, ML was part of a 4 months intensive rehabilitation programme at the Center for Rehabilitation of Brain Injury (CRBI) in Copenhagen, and he subsequently continued cognitive and speech training for some hours per week at CRBI until the end of 2009. He was described as a resourceful and highly motivated person, who has benefitted greatly from the rehabilitation efforts. At the last neuropsychological assessment performed at the CRBI in December 2009, ML was described as having normal attention and concentration, normal problem-solving abilities and normal visual processing/perception. His psychomotor tempo was difficult to evaluate because of his remaining hemiparesis. His learning and memory scores were somewhat below normal. For arithmetic, he was able to perform addition and subtraction on a normal level, but had difficulties with multiplication and division. His naming abilities had improved significantly, and he was able to name 28/30 nouns and 23/30 verbs correctly (on initial testing in 2006, he could name 1/30 and 1/30, respectively). At the time of the present investigation, ML's right arm was still paretic, while the paresis of the right leg had remitted to the degree that he can walk. His hemianopia was also remitted. ML has returned to his previous job as an auto mechanic, but now works reduced hours and performs simpler tasks than before (partly because of the arm paresis). The experimental investigation reported here was conducted in June 2011.

#### Patient MK: Wernicke's aphasia

MK is a left-handed, male native Danish speaker, who at the time of testing was 42 years old. MK suffered a haemorrhagic stroke in the territory of the left MCA in 2007. A CT scan performed one month post injury (see Figure 1) showed an intraparenchymal hemorrhage in the resorbtion phase centered on the basal ganglia, and stretching fronto-tempo-parietally in the left hemisphere. There was a central component of fresh blood measuring about 60 × 20× 40 mm, with an oedema of about 7–10 mm surrounding it. The hemorrhage stretched from the left mid temporal lobe to left centrum semovale and involved the left basal ganglia, although the caudate nucleus was spared. Cortex was also spared. The oedema affected the entire left temporal lobe, which makes the specific gyri and sulci difficult to discern. The left lateral ventricle was compressed, and the midline displaced about 3 mm toward the right. Hence the posterior part of the STS, important for audiovisual integration, may have been spared although the underlying white matter is likely to have been affected.

Initially, ML was unconscious, and spent the first 5 days in a respirator. During the first 40 days his Barthel Score increased from 0 to 49. After regaining consciousness, MK was globally aphasic and in the beginning his only response was opening his eyes when spoken to. His right arm and leg were paralytic. Soon after, he could respond to simple yes/no questions relatively correctly, and could point to some simple objects and colors. He had paralysis of the right side and a right-sided hemianopia. A logopaedic assessment two weeks post injury showed that language comprehension and production, as well as repetition were severely affected. Neuropsychological assessment, one month post injury, reported global aphasia, and problems in visuo-spatial tests. MK has been in intensive rehabilitation, first as an in-patient at Center for Neurorehabilitation—Filadelfia for six months, and later in the outpatient intensive programme at CRBI in Copenhagen for four months. A neuropsychological evaluation at CRBI in 2010 concluded that MK's visual working memory, visuo-spatial memory, and semantic processing (Pyramid and Palm trees) were relatively unaffected, while some difficulties were evident in more complex problem-solving tasks. He still suffered from moderate to severe aphasia. His confrontation naming was impaired, although greatly improved. His verbal comprehension was impaired, but he was noted to be relatively good at comprehending simple sentences. His repetition was also still impaired, as was his reading and writing. It was noted that MK often tried to write words he could not say and quite often succeeded in doing so. At the time of this investigation MK was receiving speech therapy at a community center. His left arm was still paretic, while the paralysis of the leg was remitted so that he could walk independently. MK was working part time as a practical aid at a school. The experimental investigation reported here was conducted in January 2014.

### Aphasia assessment

As part of the current investigation, ML and MK were assessed with the Danish version of the Western Aphasia Battery (WAB) (Kertesz, 1982) parts I–IV, to characterize their language abilities. Both patients provided written, informed consent according to the Helsinki declaration to participate in this study. The test results are presented in Table 1. ML's language deficit was classified as Broca's aphasia, and his Aphasia quotient was 72. MK's language deficit was classified as Wernicke's aphasia, and his Aphasia quotient was 64. The most important difference between the two patients was in the “Spontaneous speech” subsection of WAB, where, although their overall scores were almost identical, the two point difference in “Fluency” landed them on different sides of the diagnostic border between Broca's and Wernicke's aphasia according to the WAB diagnostic scoring system.

**Table 1:** **Subscores—Western Aphasia Battery**.

	**ML (Broca)**	**MK (Wernicke)**
**SPONTANEOUS SPEECH**		
Functional content	9/10	8/10
Fluency	4/10	6/10
*Total*	*13/20*	*14/20*
**COMPREHENSION**		
Yes/no questions	57/60	48/60
Auditory word recognition	59/60	51/60
Sequential commands	28/80	35/80
*Total*	*144/200*	*134/200*
**REPETITION**		
*Total*	*56/100*	*48/100*
**NAMING**		
Object naming	57/60	45/60
Word fluency	11/20	6/20
Sentence completion	6/10	8/10
Responsive speech	9/10	5/10
*Total*	*83/100*	*64/100*
***Aphasia quotient***	***72***	***64***

#### Patient ML: Broca's aphasia

At the time of the current investigation, ML's spontaneous speech was “telegraphic.” He was, however, well able to make himself understood using a combination of words and gestures. He was able to mobilize nouns and a few verbs, but did not speak in full sentences with the exception of a few common phrases like “I don't know” or “It's difficult.” As an example, in the picture description task from the WAB, ML mobilized 13 correct nouns/object names from the scene. Asked to try to describe the picture with full sentences, he added the definite article to the nouns (*a house, a man* etc.), but provided no verbs or function words. ML understood most questions and instructions either at first try or with a single repetition, and this is reflected in his relatively high score in the “Yes/no questions” and “Auditory word recognition” tests. In the more difficult “Sequential commands” test, he failed with more difficult instructions like “Use the book to point at the comb,” but interestingly managed the more “ecologically understandable” instruction “Put the pen on the book and then give it to me.” ML could repeat single words and simple sentences, but failed with sentences longer than four words. He named objects quite well, although his response-times are elevated.

In sum, ML's aphasia had remitted from Global aphasia to Broca's aphasia. He understood speech reasonably well, and his speech output was understandable but consisted mainly of nouns and very few verbs and function words.

#### Patient MK: Wernicke's aphasia

MK's spontaneous speech was fluent, and he was able to construct some full, albeit simple, sentences like “Actually I feel very good.” In the WAB picture description task, he referred to many of the objects in the picture using sentences like “There is a tree. There is also a flagpole.” Sometimes, when MK could not find the right word in speech, he could write it down correctly and then read it. He also sometimes resorted to English words when he could not find the Danish ones. In auditory verbal comprehension, he answered most questions correct (scoring 48/60), but failed relational (and grammatically challenging) questions like “Is a dog larger than a horse?” and “Does July come before May?” He could point to most images and objects in the auditory word recognition task, but had problems with left-right discrimination of body parts, and with pointing to the correct fingers. He was able to follow simple commands, but failed with longer sentences, particularly if the sequence of actions to be performed did not correspond to the word sequence. He always used the objects in the order mentioned even when this was incorrect (e.g., He responded correctly to “Point with the pen to the book” but not to “Point to the book with the pen”). He could repeat sentences up to four words correctly. In object naming he made quite a few errors (45/60 correct). Many of the responses were either phonological or semantic paraphasias (“spoon” for “fork”; “brushtooth” for “toothbrush”). A few of the words he could not name orally were written correctly (e.g., toothbrush). His responsive naming was at about the same level as his naming of objects.

In sum, MK's aphasia had remitted from Global aphasia to Wernicke's aphasia. He understood speech reasonably well, and his speech output was understandable although he had word-finding difficulties and some paraphasias.

### Control participants

In order to verify that auditory and visual stimuli were identified correctly and that the audiovisual stimuli could induce the McGurk illusion in healthy subjects we also tested two male native Danish speakers (JL, age 49, and PB, age 52) with no history of neurological disorders and self-reported normal hearing. The control participants were chosen to match the gender, approximate age, and native language of the aphasic patients.

### Stimuli and procedure

The stimuli were based on video (with audio) recordings of a male talker (one of the authors, TSA) pronouncing the bi-syllabic non-words /aga/, /aba/, and /ada/. The recordings were made using a Panasonic™ AG-HMC41E video camera, which recorded video at 25 frames per second with a resolution of 1920 × 720 pixels and audio at 44.1 kHz via an external DPA™ d:fine 4066-F microphone headset, which was not visible in the video recordings. The video was converted to QuickTime™ files with a resolution of 640 × 360 pixel to ensure efficient playback. Stimuli were presented using Matlab™ and the Psychophysics Toolbox Version 3 (Brainard, 1997; Pelli, 1997; Kleiner et al., 2007) on a MacBook™ Pro laptop computer and a pair of Genelec™ 6010A active speakers.

In each experiment, the subject was seated with his ears approximately 57 cm from the display. This was done by holding a length measure so that it extended from the plane of the speakers toward the subject and instructing the subject to adjust his position so that his ears were 57 cm from the speakers. The participants were observed to ensure that they did not move their head substantially but their position was not restrained during the experiment. The sound level was approximately 56 dB(A) measured using a sound level meter at the approximate location of the participant's head. This rather low sound level was chosen because the McGurk illusion is stronger when the sound intensity is low (Sekiyama and Tohkura, 1991; Andersen et al., 2001).

**Figure 2:**
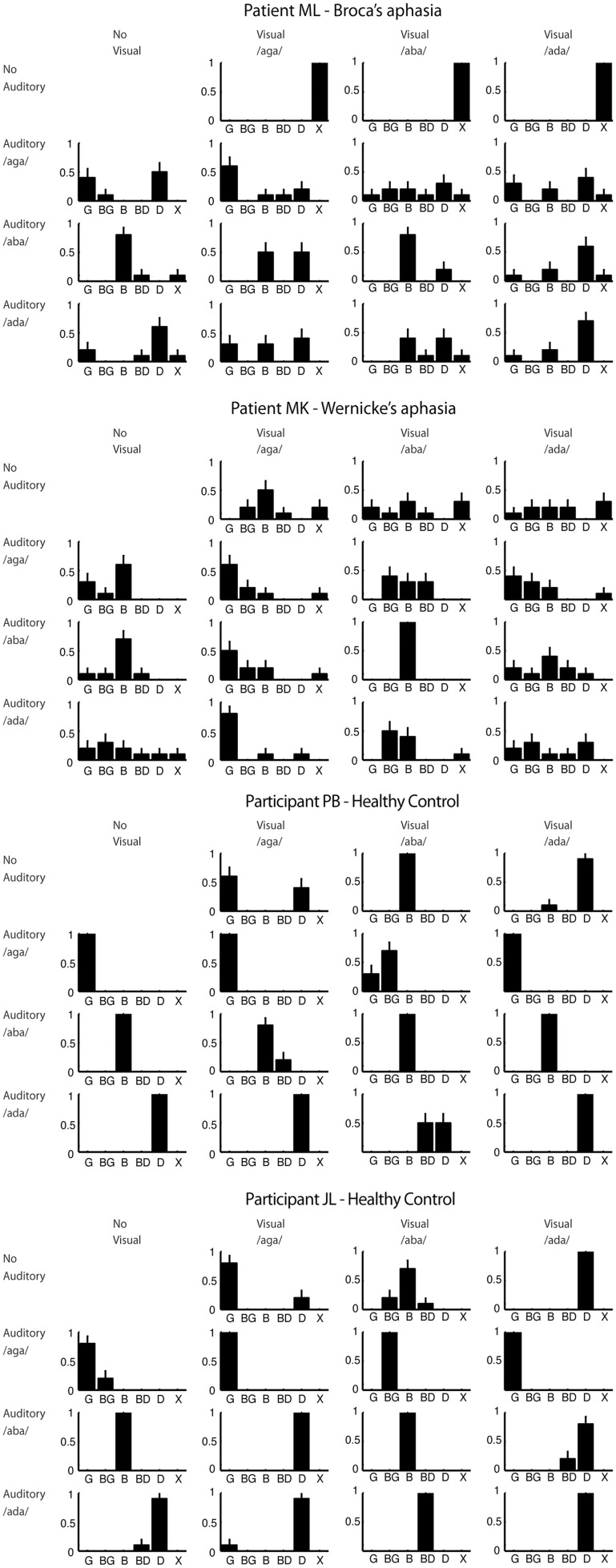
**Response proportions for all participants**. Plots are arranged in rows and columns according to the visual and auditory component of the stimulus respectively. The response category “X” corresponds to no answer. Error bars show the multinomial standard deviation.

The stimuli were auditory, visual, or audiovisual. Auditory stimuli were presented with a still photograph of the talker's face visible. Visual stimuli were presented with no sound and the task was to lip-read. Audiovisual stimuli consisted of video and sound recordings presented synchronously. The sound and video could either be one of the three natural congruent combinations or any of the six possible incongruent combinations (cf. Figure 2) of the auditory and visual stimuli. The incongruent combinations were created in Final Cut Pro by dubbing the incongruent acoustic speech recording onto the video aligning the consonant burst of the incongruent acoustic speech recording with the original congruent acoustic speech recording.

After a stimulus had played the response options “B,” “BG,” “G,” “BD,” and “D” were shown in large letters on the screen. The patients could respond by pointing to one of the response categories displayed on the screen, repeating what he had heard or declining to give an answer. We allowed for these different ways of responding in order to accommodate the patients who could have problems pointing to the response categories due to problems with reading, problems with repeating what they heard due to apraxia and problems giving any qualified guess due to their disorder. Both patients felt most comfortable using a combination of pointing and repeating when responding. The experimenter sitting next to the patient was watching his mouth movements and recorded the answer. The control participants felt most comfortable typing their responses on a keyboard.

In order to accustom the participants to the experimental setting and evaluate whether they were able to perform the experiment at all we conducted a number of training blocks containing one presentation of each stimulus in random order. The first part of the training contained only the congruent audiovisual stimuli. Both patients felt confident with the task after a single block. The next part of the training contained only the auditory stimuli. ML required three blocks and MK required two blocks before feeling confident about the task. The final part of the training contained only the visual stimuli. ML was certain that he was unable to identify any of the visual stimuli after two blocks whereas MK felt confident about the task after a single block. Performance in the training blocks was not perfect for either of the two patients. The healthy control participants were confident about the task after a single block for auditory, visual and audiovisual stimuli and their performance was perfect. After the training we proceeded to the actual experiment in which 10 repetitions of each of the auditory, visual, and audiovisual stimuli were presented in random order. The participants were instructed to look at the mouth and to report what they heard when they heard a voice (in auditory and audiovisual stimuli) but lip-read when there was no voice (in visual-only stimuli).

## Results

The response proportions for all participants are shown in Figure 2 from which it can be seen that both patients showed great variability in their responses compared to the healthy control participants. This could indicate that the patients found the task difficult in general.

Visual speech perception was particularly poor for the patients. Patient ML refused to venture a guess, which may reflect an inability to lip-read but could also reflect a lack of confidence in his own ability. Patient MK's lip-reading was also poor at 10% correct overall and poor even for /aba/ featuring a visually distinct bilabial closure. The percentage correct was 83% for each of the control participants showing that the articulations in the videos were sufficiently clear to be lip-read.

For incongruent audiovisual stimuli, in which the visual stimulus is a bilabial closure, the typical McGurk illusion is a combination illusion of hearing the bilabial closure followed by the auditory stimulus (e.g., auditory /ada/ + visual /aba/ = /abda/). This illusion was perceived by the two control participants, JL and PB respectively, in 100% and 60% of the trials including those stimuli. Only 20% of Patient ML's responses to the two incongruent audiovisual stimuli with a bilabial visual component fell in the combination response categories. For Patient MK, this number was 60%. This difference could indicate that audiovisual integration was weaker in ML for these stimuli. However, as the strength and type of McGurk illusion varies between healthy observers the difference observed here could also be due to normal variation between observers unrelated to patients' type of aphasia.

Incongruent audiovisual stimuli in which the acoustic stimulus is /b/ often create strong fusion and/or visual dominance illusions. For auditory /b/ with visual /g/ the typical McGurk illusion is a fusion illusion of hearing /d/ although a visual dominance illusion of hearing /g/ also occurs (MacDonald and McGurk, 1978; Andersen et al., 2009). Auditory /b/ with visual /d/ typically leads to a visual dominance illusion of hearing /d/ although /g/ responses have also been reported (MacDonald and McGurk, 1978). Somewhat surprisingly, whereas control participant PB did not experience these illusions for these stimuli at all, participant JL perceived them in 90% of the trials. This discrepancy is, however, not unusual as there is great inter-individual variability in the strength of the McGurk illusion (Magnotti and Beauchamp, 2014). For these two stimuli 40% of ML's responses fell in the visual dominance and fusion response categories. The same number for MK was 60%.

For audiovisual stimulus combinations of /aga/ and /ada/ (either visual /aga/ with auditory /ada/ or vice versa) observers often do not experience a McGurk illusion but respond according the auditory component of the stimulus (MacDonald and McGurk, 1978). We initially included these stimuli in the experiment for completeness although they arguably do not bare much evidence for or against our hypothesis. In fact, as the two healthy control participants did not experience the McGurk illusion for these stimuli, including them in a pooled analysis may actually lead to an under-estimation of the strength of the McGurk illusion in the patients. We therefore conducted a pooled analysis omitting these stimuli.

In order to test our main hypothesis, whether ML experience the McGurk illusion, with some statistical power we first pool responses according to three stimulus types (incongruent audiovisual, auditory, and congruent audiovisual) and two response categories (correct and incorrect according to the auditory stimulus). If an observer is influenced by visual speech in the audiovisual conditions the proportion correct should be lower in the incongruent conditions compared to the congruent conditions. Additionally, we would expect the proportion correct in the auditory condition to be higher than in the incongruent condition and lower than in the congruent condition although this latter effect is sensitive to ceiling effects (Sumby and Pollack, 1954).

**Figure 3:**
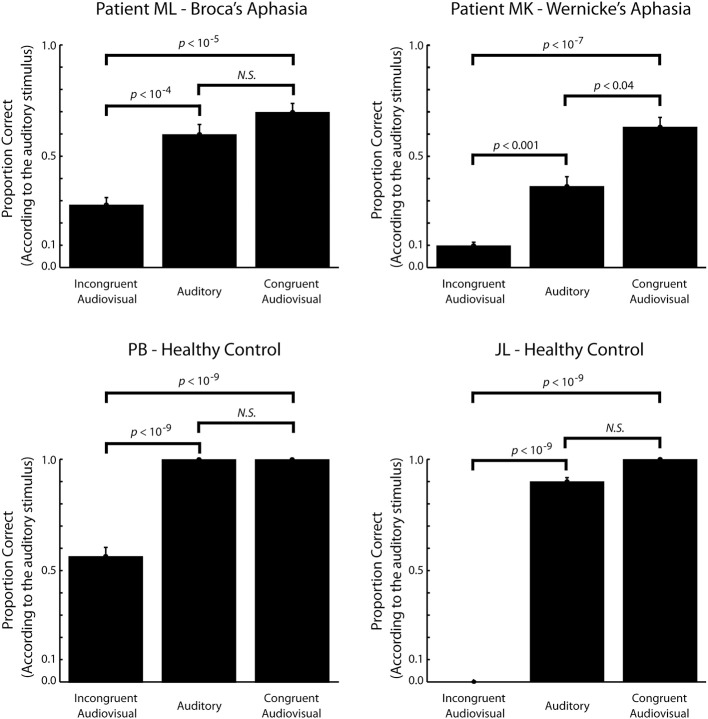
**The proportion correct (according to the auditory stimulus) as a function of stimulus type**. For the incongruent audiovisual stimuli, a low proportion correct reflects a strong McGurk illusion. Only stimuli expected to elicit visual dominance, fusion or combination illusions are included in the incongruent audiovisual stimulus category (see text for details). Within subject comparisons are based on Fisher's exact one-sided test. Error bars show the binomial standard deviation.

Figure 3 shows the proportion correct (according to the auditory stimulus) of the three stimulus types. Both patients showed a significantly higher proportion correct for congruent audiovisual stimuli than for incongruent audiovisual stimuli (*p* < 10^−5^ for patient ML, *p* < 10^−7^ for patient MK, one-sided Fisher's exact test). The proportion correct for auditory stimuli was also higher than for incongruent audiovisual stimuli (*p* < 10^−4^ for patient ML, *p* < 10^−3^ for patient MK, one-sided Fisher's exact test). This shows that both patients where influenced by visual speech. These comparisons were also highly significant (*p* < 10^−9^ for all tests, one-sided Fisher's exact test) for the control participants. For patient MK the proportion correct for congruent audiovisual stimuli was also significantly higher than the proportion correct for auditory stimuli (*p* < 0.04, one-sided Fisher's exact test). This difference was not significant for patient ML. It was also not significant for the control participants, which is probably due to ceiling effects only.

## Discussion

The sight of the talking face influenced speech perception significantly in Patient ML with Broca's aphasia. This was evident as a smaller proportion correct for the incongruent audiovisual stimuli relative to the auditory stimuli. This indicates that ML experienced a McGurk illusion and, hence, that an intact Broca's area is not required for audiovisual integration of speech. A similar effect was found in Patient MK and the healthy control participants.

In addition to the McGurk illusion, patient MK with Wernicke's aphasia showed a congruency effect evident as significantly larger proportion correct for congruent audiovisual stimuli relative to auditory stimuli. Subject ML and the two healthy control participants did not show a similar effect. For the two healthy control participants this is due, at least partly, to a ceiling effect as they were nearly perfect in identifying the auditory stimuli leaving little room for improvement. In comparison, MK, showed very poor performance for auditory stimuli, which is in good agreement with his aphasia being receptive. For subject ML we cannot exclude a ceiling effect as, perhaps, his performance for this task is capped at a fairly low level due to his injury in general. On the other hand, we cannot exclude that this somehow reflects a more subtle effect of his injury to Broca's area more specifically.

The percentage correct in the lip-reading task was very low for both patients. Although poor lip-reading skills in aphasic patients have been reported previously (Youse et al., 2004; Klitsch, 2008; Hessler et al., 2012) this is surprising given the effect that visual speech had on auditory speech perception in both patients. This could imply a dissociation between visual and audiovisual speech, which could be due to conscious lip-reading taking place in a neural pathway, which is different from the one that affects auditory processing (Bernstein and Liebenthal, 2014). A similar dissociation was found in a study showing that point-light visual speech can cause a McGurk illusion even when observers are unaware of its speech-like nature and, hence, unable to lip-read (Rosenblum and Saldaña, 1996). This, to some degree, mimics how the patients seemed to perceive visual and audiovisual speech in the current study. Rosenblum and Saldaña (1996) also showed that static faces generally do not cause a McGurk effect even though they can be lip-read to some degree. Hence, where visual speech perception can be based on both static and dynamic information, only dynamic visual information is integrated with acoustic features. In support of this dissociation Calvert and Campbell (2003) found that lip-reading static faces activate different cortical areas than does lip-reading of dynamic features. As static natural images contain both configurational and featural facial information while point-light speech contains only configurational information another possible dissociation would be that visual speech perception can be based on both configurational and featural information while only featural information is integrated audio-visually. Both of these functional dissociations can be related to dual-stream anatomical models where the dorsal pathway relays mainly dynamic/featural information while the ventral pathway relays mainly static/configuration information (Bernstein and Liebenthal, 2014). Although this picture might not reflect the true complexity of the underlying neural structure (Bernstein and Liebenthal, 2014) it serves to show that it is not unlikely that lesions causing aphasia can influence visual and audiovisual speech perception differently.

Overall, performance in both patients was quite variable with many atypical responses. This variability matches that seen in previous studies of audiovisual speech perception in aphasic patients (Campbell et al., 1990; Youse et al., 2004; Klitsch, 2008; Hessler et al., 2012). These studies generally agree that aphasics, in general, do experience the McGurk illusion and thus integrate auditory and visual information in speech perception. They also agree that aphasics generally have poor lip-reading skills. Only few of them attempted a distinction between subtypes of aphasia and none have reached a conclusion on the specific role of Broca's aphasia.

A number of studies have previously examined audiovisual integration of speech in aphasic patients. Campbell et al. tested a patient that was diagnosed with aphasia after a cardiovascular accident (CVA) in the left MCA (Campbell et al., 1990). At the time of testing he was a fluent speaker experiencing that speech “sounded funny” indicating that his aphasia was mild and mainly receptive. He was also slightly dyslexic. This patient's performance was fairly poor (<50% correct) in a consonant identification task but the patient seemed to experience visual dominance and fusion illusions for incongruent audiovisual stimuli and Campbell et al. concluded that he was influenced by visual speech in his performance. This is in agreement with his lip-reading ability, which, for words, fell within the normal range.

Youse et al. tested a patient with mild anomic aphasia based on the Western Aphasia Battery, after a CVA affecting the territory of the MCA not unlike patient ML in the current study (Youse et al., 2004). Apparently, this patient showed a strong McGurk effect for auditory /bi/ with visual /gi/, which he perceived as /di/. Youse et al. did however note that this finding could be influenced by a strong response bias toward /di/ apparent in the auditory only condition. For auditory /gi/ with visual /bi/ they did not observe the typical combination illusion of perceiving /bgi/ but, in our opinion, a fairly strong visual dominance illusion of perceiving /bi/. Youse et al. concluded that the results did not show clear evidence of audiovisual integration in this patient. Notably, this patient's ability to lip-read was also very poor.

Klitsch (2008) investigated the McGurk effect in a group of six aphasic patients of which three were diagnosed with Broca's aphasia and the other three were diagnosed with Wernicke's aphasia. She found that the aphasic group experienced the McGurk illusion but did not distinguish between the types of aphasia. The proportion correct for lip-reading was 50% for this group given three response categories.

Hessler et al. (2012) investigated audiovisual speech perception in three aphasics and an age-matched control group. The three aphasics, were diagnosed as Wernicke's, anomic and mixed respectively. The mixed aphasic had suffered an ischaemic CVA in the left MCA, as our patient ML, and showed audiovisual interactions by giving more correct responses to congruent audiovisual speech than to auditory speech. This participant was tested on one incongruent combination of auditory and visual phonemes, auditory /p/ and visual /g/, and seemed to perceive the McGurk illusion in 61% of the experimental trials as measured by the proportion of incorrect responses. However, this should be compared to an error rate of 45% in the auditory condition—an error rate which was averaged across three different stimulus types, /k/, /p/, and /t/. Hessler et al. did not directly compare how well auditory /p/ was perceived when presented acoustically and when presented audiovisually, dubbed onto visual /k/. Hessler et al. concluded that none of the aphasic participants showed a preference for McGurk-type answers. The lip-reading performance for the three aphasic patients was, accordingly, poor ranging from 24 to 52% correct given only three response categories. For the control group, the corresponding range was 67–93%.

These studies by Hessler et al. and Klitsch suffered from very weak McGurk illusions (as low as 20% in Klitsch' study) even in the healthy control groups. Both studies ascribe this to a language effect specific to the Dutch language in which the studies were conducted. This makes it difficult to interpret whether the results in the aphasic group are representative.

Based on the studies described above, the poor lip-reading skills and atypical McGurk illusions that we found in Patient ML with Broca's aphasia seem to fall within the range generally seen in the aphasic population. Therefore, they cannot be ascribed to his lesion in Broca's area specifically. The variability seen across participants in these studies is not specific to aphasic patients but is also seen across studies of healthy subjects (Colin and Radeau, 2003). It is likely to be due, not only to variation across participants but also to variation across stimuli (Magnotti and Beauchamp, 2014). Hence, specific differences that are statistically significant are likely to be seen even between healthy participants and, to our knowledge, the reasons for this are unknown. Statistical comparisons of specific differences between aphasic patients or between patients and healthy controls will therefore be difficult to interpret without larger populations (Saalasti et al., 2011a,b; Meronen et al., 2013). Therefore, rather than conducting statistical analyses between participants, we limited our analysis to showing that audiovisual integration did take place in a patient with Broca's aphasia.

In accordance with our findings, Fridriksson et al. (2012) recently showed that speech production in Broca's aphasics can improve dramatically when they shadow audiovisual speech. Notably, this effect does not occur for auditory or visual speech. This shows that Broca's aphasics have some ability to integrate auditory and visual speech for use in speech production but the study does not address whether it also influences speech perception in general. As speech perception and production have been suggested to receive information from two, anatomically distinct, parallel streams (Hickok and Poeppel, 2007) the perceptual McGurk effect in Broca's aphasics may be due to a different mechanism than that studied by Fridriksson et al.

In summary, our findings show that speech perception in a patient with Broca's aphasia in influenced by the sight of the talking face as this patient experiences a McGurk illusion, which, although somewhat atypical, is little different from the McGurk effect seen in the aphasic population in general. This offers no confirmation of the hypothesis that Broca's area should be necessary for audiovisual integration of speech, contrary to Ramachandran et al.'s (1999) preliminary findings, and hence, no confirmation of the strong mirror neuron MT. Whether a lesion in Broca's area can have a more subtle effect on audiovisual integration, as it has on auditory speech perception, cannot be ruled out by the current results. Therefore, we do not consider our findings in disagreement with recent, more moderate versions of the MT in which articulatory representations have a subtle role in speech perception and are located in a distributed network of brain structures.

### Conflict of interest statement

The authors declare that the research was conducted in the absence of any commercial or financial relationships that could be construed as a potential conflict of interest.
